# A Global Perspective on the Emerging Threat of Zika and Dengue Co-infection

**DOI:** 10.1371/journal.pntd.0014257

**Published:** 2026-04-29

**Authors:** Arnob Saha, Mahir Anjum, Shyrin Jebun Labiba, Mirza Jaman, Abdullah Al Sayed, Rafsun Jany Rahat

**Affiliations:** 1 Department of Microbiology and Hygiene, Faculty of Veterinary Science, Bangladesh Agricultural University, Mymensingh, Bangladesh; 2 Department of Pharmacology, Faculty of Veterinary Science, Bangladesh Agricultural University, Mymensingh, Bangladesh; 3 Department of Genetic Engineering and Biotechnology, University of Chittagong, Chittagong, Bangladesh; 4 Department of Biochemistry and Molecular Biology, Gopalganj Science and Technology University, Gopalganj, Bangladesh; 5 Faculty of Animal Husbandry, Bangladesh Agricultural University, Mymensingh, Bangladesh; University of Cambridge, UNITED KINGDOM OF GREAT BRITAIN AND NORTHERN IRELAND

Dengue virus (DENV) and Zika virus (ZIKV) share the mosquito vector *Aedes aegypti* and together represent an increasing global public health threat. This risk has become tangible, as evidenced by Bangladesh’s first confirmed DENV–ZIKV co-infections during the 2023 dengue outbreak, marking a significant escalation in arboviral risk within the country [1]. Comparable tropical climates, rapid urbanization, and inadequate vector control systems are present in many regions, creating conditions where co-infection is increasingly probable. This scenario requires immediate attention at both national and international levels.

The co-circulation of DENV and ZIKV has repeatedly resulted in documented co-infections, as illustrated during the 2015–2016 ZIKV epidemic in the Americas. Enhanced surveillance in northeastern Brazil identified arboviral co-infections, including DENV/ZIKV, and demonstrated that presumptive clinical diagnosis was frequently inaccurate in settings of co-circulation [2]. Similarly, molecular surveillance at the Colombian–Venezuelan border confirmed simultaneous circulation of DENV, ZIKV, and chikungunya virus (CHIKV), with measurable DENV/ZIKV co-infection prevalence [3]. Beyond clinical reports, experimental evidence suggests co-infection can modify transmission potential: co-infection of DENV and ZIKV in *Aedes aegypti* increased viral replication relative to single infections in at least some experimental settings [4], and sequential co-infection experiments have shown altered (biased) virus transmission dynamics from mosquitoes [5].

The Asia-Pacific region has also experienced such events, with early human DENV/ZIKV co-infections documented in New Caledonia in 2014 [6]. In Southeast Asia, surveillance work in Thailand has similarly identified co-infections among circulating arboviruses, reinforcing that co-infection risk is widespread rather than isolated [7].

Co-infection presents significant challenges for patient management, primarily due to diagnostic difficulties. ZIKV and DENV can produce overlapping symptoms (e.g., fever, rash, and myalgia), and clinical differentiation is unreliable in co-circulation settings [2,8]. Serological assays can be problematic because flavivirus cross-reactivity may produce misleading results [9]. Molecular methods (e.g., multiplex RT-PCR) improve specificity for differential diagnosis [10], but access is often concentrated in urban laboratories, leaving many co-infections undetected and obscuring the true disease burden.

A major concern involves the clinical implications of co-infection. ZIKV is strongly associated with neurological complications, including Guillain-Barré syndrome, and congenital infection can cause severe fetal outcomes; these risks complicate prognosis and management when DENV and ZIKV occur together [8].

Effective response requires coordinated global action. Strengthening vector control, the foundation of prevention is essential. Innovative and sustainable strategies, such as releasing *Wolbachia*-infected mosquitoes, have demonstrated the capacity to reduce arboviral transmission and disease burden [11]. In parallel, health systems should upgrade laboratory capacity for differential diagnosis, train clinicians for co-circulation/co-infection contexts, and update national guidelines to explicitly address co-infection management. Sentinel surveillance systems can enable earlier detection of co-circulation and co-infection signals, particularly in high-risk urban settings. A comprehensive Table 1 summarizes available epidemiological reports of arbovirus co-infection, including key demographic characteristics such as age, gender distribution, country/region, outbreak setting, and clinical outcomes where reported.

**Table 1:** Summary of reported arbovirus co-infection events (DENV, ZIKV, CHIKV).

Virus combination	Country/Region	Year	Setting	co-infected cases (% of tested)	Age (median/range)	Gender (% female)	Pregnant (cases)	Key outcomes	Reference
DENV–ZIKV	Bangladesh (Dhaka)	2023	Dengue outbreak investigation	1/152 (0.66%) co-infected; 4/152 (2.6%) ZIKV mono-infection	Median 24 years	0% (all males)	0	Mild febrile illness; all recovered; tightly clustered within 1 km radius	[1]
DENV–CHIKV–ZIKV (triple) and all pairwise combinations	Colombian–Venezuelan border	2015–2016	Febrile syndrome cohort (157 samples)	DENV/CHIKV 7.64%; DENV/ZIKV 6.37%; CHIKV/ZIKV 5.10%; triple 1.91%	Mean age higher in ZIKV (29.7 years) than DENV/CHIKV (21.1 years)	Not specified	Not specified	All ambulatory; no specific increase in severity reported	[3]
DENV–ZIKV	New Caledonia	2014	Outbreak sentinel surveillance	2	14-year-old boy; adult woman	Not applicable	0	Mild, nonhospitalized, full recovery	[6]
DENV–CHIKV–ZIKV (triple)	Nicaragua	2015–2016	Febrile surveillance in children	1 triple infection (plus multiple pairwise co-infections)	Children, median 7–8 years	Approximately half female	Not specified	Clinical course similar to mono-infections	[12]
CHIKV–ZIKV	Colombia (national)	2015–2016	National surveillance (suspected ZIKV cases)	28/23,871 (0.12%)	28 years (IQR 21–39)	73.50%	14	5 adult deaths, 2 fetal deaths	[13]
DENV–CHIKV	Colombia (national)	2015–2016	National surveillance	3/23,871 (0.01%)	54–71 years	0%	0	2 deaths (neurologic/hepatic failure)	[13]
DENV–CHIKV	Cali, Colombia	2015	Outbreak case report	1	Adult	Not specified	0	Fatal, severe hepatic and cardiac involvement	[14]

Co-infections between dengue, Zika, and other arboviruses likely arise from shared Aedes vectors, overlapping ecological drivers, and direct viral–viral interactions within mosquitoes and human hosts, which together can modulate transmission and disease expression [4,13]. Co‑infection experiments in *Aedes aegypti* demonstrate that simultaneous dengue and Zika virus infection can mutually enhance viral replication and increase vector competence compared with single infections, suggesting that co‑infected mosquitoes may more efficiently transmit one or both viruses [4]. At the host level, dengue and Zika viruses share extensive antigenic similarity, and preexisting cross‑reactive antibodies can bind but fail to fully neutralize the second virus, leading to antibody‑dependent enhancement and increased infection of Fcγ receptor–bearing cells in vitro and in vivo [15–18]. These reciprocal immune‑enhancement phenomena raise concerns that co-infection or closely spaced sequential infections could exacerbate viremia, inflammatory responses, and potentially neurological or congenital complications, although the magnitude of this risk at the population level remains uncertain [15–18]. Clinically, arboviral co-infections are uncommon but well documented in surveillance and case‑series data, and they have been associated with both mild febrile illness and severe outcomes such as multiorgan failure and adverse fetal events, highlighting the need for multiplex molecular diagnostics, careful interpretation of serology in co‑circulation settings, and consideration of potential enhancement effects when designing and evaluating future dengue and Zika vaccines or antibody‑based interventions [13,19,20].

**Fig 1:**
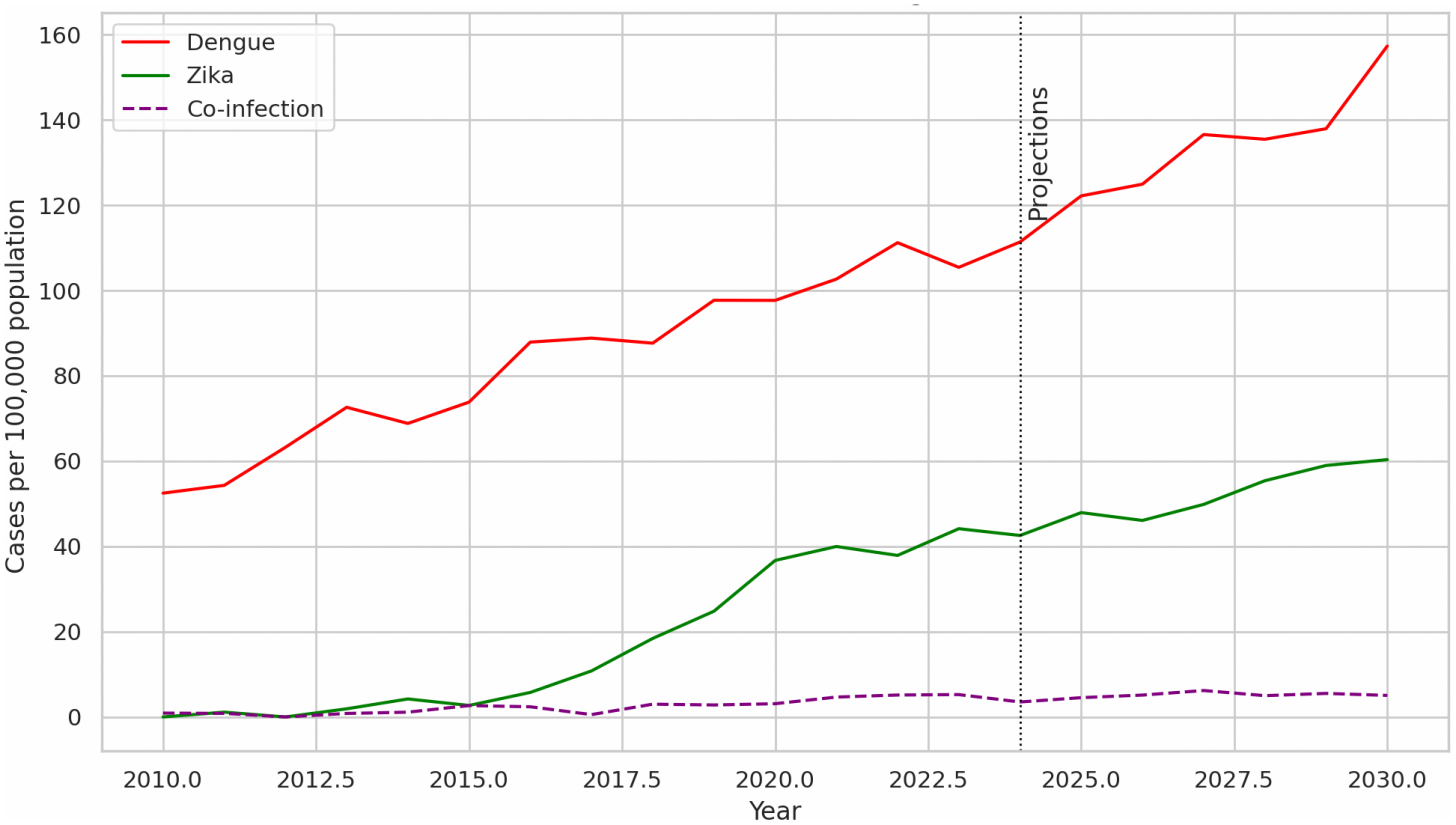
Hypothetical, historical, and future trends of Dengue infection, Zika infection, and Dengue-Zika Co-infection.

The emergence of ZIKV-DENV co-infections signifies a complex new phase in arboviral control. A comprehensive, multilayered response is essential, encompassing enhanced surveillance, accurate diagnostics, robust vector control, and active community engagement. Without coordinated action, in future, the combined morbidity and health-system strain associated with dual viral risks will exceed current public health capacities (Fig 1).
